# Assessment of Paracetamol Toxic Effects under Varying Seawater pH Conditions on the Marine Polychaete *Hediste diversicolor* Using Biochemical Endpoints

**DOI:** 10.3390/biology11040581

**Published:** 2022-04-11

**Authors:** David Daniel, Bruno Nunes, Edgar Pinto, Isabel M. P. L. V. O. Ferreira, Alberto Teodorico Correia

**Affiliations:** 1 Departamento de Biologia, Campus de Santiago, Universidade de Aveiro (UA), 3810-193 Aveiro, Portugal; d.daniel@ua.pt; 2 Centro Interdisciplinar de Investigação Marinha e Ambiental (CIIMAR/CIMAR), Terminal de Cruzeiros do Porto de Leixões, Av. General Norton de Matos S/N, 4450-208 Matosinhos, Portugal; atcorreia.ciimar@gmail.com; 3 Centro de Estudos do Ambiente e do Mar (CESAM), Campus de Santiago, Universidade de Aveiro, 3810-193 Aveiro, Portugal; 4 Escola Superior de Saúde (ESS) do Instituto Politécnico do Porto (IPP), Rua Dr. António Bernardino de Almeida 400, 4200-072 Porto, Portugal; ecp@ess.ipp.pt; 5 LAQV/REQUIMTE-Laboratório de Bromatologia e Hidrologia, Departamento de Ciências Químicas, Faculdade de Farmácia, Universidade do Porto, 4200-465 Porto, Portugal; isabel.ferreira@ff.up.pt; 6 Faculdade de Ciências da Saúde da Universidade Fernando Pessoa (FCS/UFP), Rua Carlos da Maia 296, 4200-150 Porto, Portugal; 7 Instituto de Ciências Biomédicas Abel Salazar da Universidade do Porto (ICBAS), Rua de Jorge Viterbo Ferreira 228, 4050-313 Porto, Portugal

**Keywords:** pharmaceuticals, climate change, bioassay, biomarkers, oxidative stress

## Abstract

**Simple Summary:**

Context of climate change is being widely studied, nevertheless its effects in the toxicity of other contaminants have been poorly study. Particularly, the effects of ocean acidification on the modulation of pharmaceutical absorption and consequent effects, have not been extensively addressed before. In this study, we aimed to assess the effects of ocean acidification (specifically pH values of 8.2, 7.9, and 7.6) combined with paracetamol exposure (0, 30, 60, and 120 µg/L) on the polychaeta Hediste diversicolor. To do so, specific biomarkers were measured namely (CAT), glutathione S-transferases (GSTs), acetylcholinesterase (AChE), and cyclooxygenase (COX) activities, as well as thiobarbituric acid reactive substance (TBARS), were quantified to serve as ecotoxicological endpoints. Alterations of CAT, and GSTs activities, and TBARS levels indicate an alteration in redox balances. Differences in exposed pH levels indicate the possible modulation of the absorption of this pharmaceutical in ocean acidifications scenarios. Alterations in AChE were only observed following paracetamol exposure, not being altered by media pH. Hereby obtained results suggest that seawater acidification is detrimental to marine wildlife, since it may enhance toxic effects caused by environmental realistic concentrations of pharmaceuticals. This work is crucial to understand the potential effects of pharmaceuticals in a climate change scenario.

**Abstract:**

Increasing atmospheric carbon dioxide (CO_2_) levels are likely to lower ocean pH values, after its dissolution in seawater. Additionally, pharmaceuticals drugs are environmental stressors due to their intrinsic properties and worldwide occurrence. It is thus of the utmost importance to assess the combined effects of pH decreases and pharmaceutical contamination, considering that their absorption (and effects) are likely to be strongly affected by changes in oceanic pH. To attain this goal, individuals of the marine polychaete *Hediste diversicolor* were exposed to distinct pH levels (8.2, 7.9, and 7.6) and environmentally relevant concentrations of the acidic drug paracetamol (PAR: 0, 30, 60, and 120 µg/L). Biomarkers such as catalase (CAT), glutathione S-transferases (GSTs), acetylcholinesterase (AChE), and cyclooxygenase (COX) activities, as well as peroxidative damage (through thiobarbituric acid reactive substance (TBARS) quantification), were quantified to serve as ecotoxicological endpoints. Data showed a general increase in CAT and a decrease in GST activities (with significant fluctuations according to the tested conditions of PAR and pH). These changes are likely to be associated with alterations of the redox cycle driven by PAR exposure. In addition, pH levels seemed to condition the toxicity caused by PAR, suggesting that the toxic effects of this drug were in some cases enhanced by more acidic conditions. An inhibition of AChE was observed in animals exposed to the highest concentration of PAR, regardless of the pH value. Moreover, no lipid peroxidation was observed in most individuals, although a significant increase in TBARS levels was observed for polychaetes exposed to the lowest pH. Finally, no alterations of COX activities were recorded on polychaetes exposed to PAR, regardless of the pH level. The obtained results suggest that seawater acidification is detrimental to marine wildlife, since it may enhance toxic effects caused by environmental realistic concentrations of acidic drugs, such as PAR. This work was crucial to evidence that ocean acidification, in the context of a global change scenario of increased levels of both atmospheric and oceanic CO_2_, is a key factor in understanding the putative enhanced toxicity of most pharmaceutical drugs that are of an acidic nature.

## 1. Introduction

Due to the continuous release of carbon dioxide (CO_2_), mostly from anthropogenic sources, such as the manufacture of cement and electricity production, through the burning of fossil fuels [1,2,3], CO_2_ atmospheric concentration has systematically increased in recent years [1,4,5]. Moreover, CO_2_ levels increased simultaneously in the oceans after its dissolution in seawater, with the formation of carbonic acid [6]. This weak acid tends to be partially neutralized by the buffering capacity of seawater due to the role of photoautotrophic organisms, namely cyanobacteria and algae [7]. Nevertheless, this buffering capacity is limited, and the excess of carbonic acid built up in the oceans has already lowered, on average, the seawater pH by about 0.1 units since the industrial revolution [8,9]. Oceans have a pH value of approximately 8.2 [10], and it is estimated that this value will decrease by as much as 0.4 units by the end of the twenty-first century [11] and as much as 0.7 units by 2300 [12]. These changes will necessarily lead to serious environmental consequences, including imbalances in the carbon cycle [7], the increased leaching of metals, and changes in the solubility profiles of most marine contaminants [13]. This will happen since the majority of these chemicals have weak acidic or basic natures, and their equilibria in water are modulated by the extent of non-ionized vs. ionized forms, which is strongly conditioned by pH. This chemical equilibrium is a key factor for the absorption of most xenobiotics by biota, since electrically charged (ionized) forms are not likely to be promptly absorbed by biota, while non-ionized forms tend to easily cross biological membranes [14].

Among the chemicals that are prominent contributors for this scenario, pharmaceuticals are widely used in human medicine and veterinary practices as well as agriculture and aquaculture activities, being thus considered as environmental emerging contaminants [15]. Consequently, these substances may end up in the environment through human or animal excretion, by processes of leaching, or by direct disposal, among other processes [16]. Pharmaceuticals are characterized for their moderate lipophilic nature, strong biological activity, the potential to bioaccumulate, environmental pseudo-persistence, and resistance to most common treatment processes at wastewater treatment plants (WWTPs) [17,18,19]. Despite their origin, pharmaceuticals, in general, can occur in the water compartment in their native form or as metabolites [20,21], being detected in concentrations ranging from ng/L to μg/L [20,22]. This set of characteristics can make them deleterious compounds towards non-target species, especially those from the aquatic compartment [19,23]. Their ubiquitous presence in the water compartment and the fact that their solubility and absorption rates are strongly conditioned by environmental pH are factors to be considered when studying their toxicity.

Nonsteroidal anti-inflammatory drugs (NSAIDs) are a class of pharmaceuticals whose presence has been consistently reported in water samples [19,20]. The general mechanism of action of this class is based on the inhibition of the enzyme cyclooxygenase (COX), which prompts these drugs to be used as anti-inflammatory agents, by preventing prostaglandins (PGs) and thromboxane biosynthesis [20,24]. This pharmacological activity may, however, interfere with other physiological processes that are controlled or modulated by PGs in non-target organisms, such as reproduction, osmoregulation, and immune response [25]. This therapeutic class is globally important, since it corresponds, worldwide, to one of the most used class of drugs, and it includes substances such as diclofenac, acetylsalicylic acid, ibuprofen, and naproxen; despite not being a classic NSAID, paracetamol (PAR) shares some of the critically important therapeutic properties of this class of drugs, being among the most used pharmaceuticals in the world [20,26]. PAR is a widely used analgesic and antipyretic drug [27], being considered a safe drug at therapeutic dosages [28]. PAR has been found in wastewater effluents, surface waters, and marine environments in concentrations in the µg/L range [29,30,31,32]. Previous studies have shown that environmentally realistic concentrations of PAR could cause deleterious effects in aquatic non-target organisms, such as oxidative stress [33,34,35], neurotoxicity [34,36,37], impacts on physiological processes [38,39], and reductions in total offspring [34]. It has been well documented that pH affects the absorption rate of pharmaceuticals (e.g., when in water, weak acidic molecules are better absorbed in acid media [40,41,42]). Despite being a generic principle that is especially valid for simple processes (cellular and organ levels), this seems also to be valid for the environmental processes of absorption of toxicants from external media. Previous studies have shown the effect of pH on the absorption and toxicity of several contaminants [43,44]. Consequently, and assuming the characteristics of PAR (which has been classified as a weak acid [45]), lower pH values may enhance the absorption (and the toxicity) of this drug. This constitutes a highly relevant hypothesis in a scenario of global change because of a shift in pH values (as predicted to occur in the next decades) and can lead to an increased ecotoxicological risk towards non-target organisms.

Hediste diversicolor, commonly known as the ragworm, is a polychaete of the family Nereidae that inhabits marine and estuarine coastal environments [46]. This aquatic worm is of high economic importance, since it is frequently used as bait in recreational fishing [46]. It is also ecologically relevant because, besides being a nutrient source for other animals such as birds, it involves the sediment, preventing hypoxia; it is also important in the nutrient and carbon cycles [47]. *H. diversicolor* is a sentinel species with a broad geographic distribution [48], showing a high tolerance to fluctuations of several abiotic factors, such as salinity, temperature, and pH [49]. These characteristics, in addition to being abundant all year around and being easy to collect and maintain under laboratory conditions, made this species successful in previous ecotoxicological and biomonitoring studies [50,51,52,53,54].

The use of biomarkers is a powerful approach to evaluate contamination effects on organisms [55]. The activities of key enzymes may signal adaptive changes elicited by the environmental exposure to xenobiotics and serve as biomarkers, being therefore used as proxies of deleterious effects caused by exposure to several xenobiotics, including pharmaceuticals and seawater acidification [56,57,58]. Furthermore, oxidative effects caused by some of these substances, where drugs are included, are likely to result in peroxidative damages in the membrane lipids of exposed aquatic organisms [37,59]. Finally, some biomarkers (e.g., acetylcholinesterase), despite not being involved in the antioxidant defence, seem also to respond to pro-oxidative conditions [34,60].

We intend to assess the effects of PAR in the polychaeta *H. diversicolor* and whether an acidification scenario may modulate the potential effects of PAR. The aim of this study was to assess the putative toxic effects of chronically exposed individuals of *H. diversicolor* to environmentally relevant concentrations of PAR under different pH levels by simulating seawater acidification scenarios based on well-established climate changes predictions [11]. We assessed key biochemical responses, namely antioxidant defence mechanisms (Catalase, CAT), phase II metabolic detoxification (Glutathione S-Transferases, GSTs), lipid peroxidation (Thiobarbituric Acid Reactive Species, TBARS), neurotoxicity (Acetylcholinesterase, AChE), and prostaglandin biosynthesis (Cyclooxygenase, COX).

## 2. Materials and Methods

### 2.1. Biological Sampling

Ragworms were hand-collected using a metal fork on December 2017 in the Douro River estuary at Reserva Natural Local do Estuário do Douro (41.139337 N, −8.655909 W), Northern Portugal, during the low tide period. The sampling area is considered unpolluted, or Class I according to the international guidelines for classification of the environmental quality of coastal sediments [61] based on the background levels for the major polycyclic aromatic hydrocarbon (PAH) classes and heavy metals [62]. After collection, the individuals were transported to the laboratory in refrigerated plastic boxes, where they were screened and selected. Only apparently healthy animals with a pre-defined mass (0.500 ± 0.100 g) were selected. These animals were then subjected to a two-week quarantine/depuration/acclimation period, according to ASTM [63]. During quarantine, animals were kept in artificial salt water (Tropic Marin® SEA SALT from Tropic Marine) at a salinity of 20 ± 2, with continuous aeration, and a temperature of 20 ± 1 ${}^{{}^{\circ}}$C. These abiotic conditions are appropriate for the maintenance of this species [64] and similar to those used in other ecotoxicological studies using the same test organism [52,53,54]. Sediment from the sampling area was previously washed with distilled water and incinerated at 500 ${}^{{}^{\circ}}$C. Animals were fed *ad libitum* three times a week with commercial fish food (Activpet™, Lisbon, Portugal), and the medium was fully renewed twice a week. Animals were kept in 50 L plastic boxes with a density of 400 individuals/m${}^{2}$ [65].

### 2.2. Chemicals and Test Concentrations

Paracetamol was purchased from Sigma-Aldrich Chemical (CAS number 103-90-2; with purity >99%). Stock solutions (4.8 mg/L, 9.6 mg/L, and 19.2 mg/L) were prepared by diluting PAR in artificial seawater. The nominal concentrations used (30, 60, and 120 μg/L) were within the range of concentrations found in the aquatic compartment (10–200 µg/L), namely in wastewater effluents and surface waters [29,30,66,67]. Nominal concentrations (including the stock solutions) were confirmed by solid-phase extraction (SPE) followed by a Liquid Chromatography with tandem Mass Spectrometry (LC-MS/MS) analysis as described by Afonso-Olivares et al. [68]. For that, acetaminophen-D4 (100 μg/mL in methanol, Cerilliant^®^, CAS Number:64315-36-2) was purchased from Supelco. Methanol (CH_3_OH, CAS number 67-56-1) and formic acid (H_2_CO_2_, CAS number 64-18-6) of HPLC grade were acquired from Sigma-Aldrich. A stock solution of 1000 mg/L paracetamol was prepared in HPLC grade methanol. A 7-level series of calibrators (from 10 to 1000 ng/mL) was prepared by proper dilution of the 1000 mg/L acetaminophen stock solution with a 20% methanol solution containing 0.1% formic acid. Other chemicals, namely those used for the preparation of buffers, standards, and protein reagents, were purchased from Biorad, MercK, Acros Organics, and PanReac.

### 2.3. SPE and LC-MS/MS Procedures

Water samples (stock solutions and tested concentrations) were extracted using the SPE method described by Afonso-Olivares et al. [68]. Briefly, SPE cartridges (Oasis HLB, 6 mL, 200 mg, Waters) were conditioned with 5 mL of methanol followed by 5 mL of ultrapure water (Milli-Q water, Merck, Millipore, Germany; 18.2 MΩ at 25 ${}^{{}^{\circ}}$C) at a flow rate of 10 mL/min. Afterward, water samples (30 mL) were loaded onto the cartridges, followed by a washing step with 5 mL of ultrapure water. The cartridges were dried under vacuum, and acetaminophen was eluted with 2 mL of methanol. The eluates were evaporated under a gentle nitrogen stream and reconstituted with 0.2 mL of 20% methanol containing 0.1% formic acid. Paracetamol analysis was performed on a Waters 2695 XE separation module coupled to a Waters Micromass^®^ Quattro micro™ API triple quadrupole mass analyzer (Waters, Manchester, UK). Chromatographic separation was achieved with a core-shell Kinetex^®^ EVO C18 column (i.d. 2.1 mm × 100 mm, particle size 2.6 µm) fitted with a SecurityGuard™ ULTRA UHPLC pre-column (C18, 3 mm × 4 mm) at a flow rate of 0.2 mL/min. The columns were kept at 35 ${}^{{}^{\circ}}$C during analysis, and the injection volume was 10 µL. Isocratic elution was used with a mobile phase consisting of 20% methanol containing 0.1% formic acid.

Quantification of paracetamol was performed in Multiple Reaction Monitoring (MRM) mode using a triple quadrupole mass spectrometer (MS/MS). The mass spectrometer was used in positive electrospray mode (ES+). The optimized MS parameters were as follows: capillary voltage: 3.0 kV; source temperature: 120 ${}^{{}^{\circ}}$C; desolvation temperature: 400 ${}^{{}^{\circ}}$C; desolvation gas flow: 600 L/h; multiplier: 650 V. High purity nitrogen (≥99.999%) and argon (≥99.999%) were used as the desolvation/cone and collision gases, respectively. MRM transitions, cone voltage, and collision energies for acetaminophen and acetaminophen-D4 were determined by flow injection analysis and are listed in Table 1. Data acquisition was performed by the MassLynx V4.1 software. Measured concentrations are within the expected nominal values (stock solutions: 4.6 ± 0.1, 9.5 ± 0.2, and 19.5 ± 0.3 mg/L; exposure solutions: 27.4 ± 0.6, 57.9 ± 1.0, and 123.7 ± 2.4 µg/L).

### 2.4. Experimental Design

**Scheme 1:**
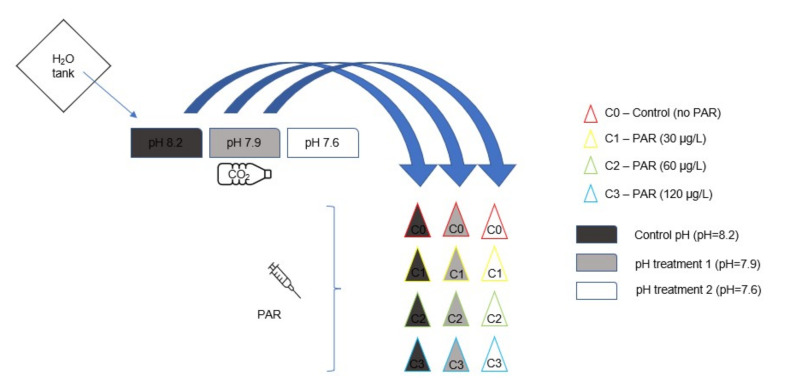
A schematic representation of the experimental set-up in which the 4 levels of organization are represented (i.e., first level sweater tank (1); second level CO_2_ mixing flasks (3); third level, contaminant mixing flasks (4); fourth level, experimental units (12).

Animals were exposed similarly to what was described in [44] and as shown in Scheme 1. Briefly, 12 L plastic boxes were used and divided into 3 compartments (pseudo-replicates), where 7 animals were exposed per compartment, for a total of 21 animals per treatment. Each box was filled with artificial seawater and clean sediment (from the sampling site) in a proportion of 1 part of sediment per 3 parts of water. The salinity was 20 ppt, and the pH was set to 8.2 (control), 7.9, and 7.6. These pH values were established based on current oceanic pH values and considering results from prediction models for seawater acidification scenarios [11,12]. Acidified water was obtained from a system similar to the one used by Cardoso et al. [69] in which pure CO_2_ was directly diffused in 3 upper containers filled with seawater. The release of CO_2_ was controlled by an Aqua Medic^®^ AT Control-SW, version 9.0 system, connected to a solenoid valve that kept pH values around the pre-established values of ±0.1, and this was checked by an independent probe every other day. Alkalinity was measured regularly along the experiment (65.3 ± 6.9 mg/L of CaCO_3_) using a Photometer YSI (9300) and Palintest alkalinity (M) tablets. Paracetamol was directly added to each box using a peristaltic pump (Gilson Miniplus 2) with a continuous flux of 0.625 µL/s from stock solutions of 4.8 mg/L, 9.6 mg/L, and 19.2 mg/L to reach the nominal concentrations of 30 µg/L, 60 µg/L, and 120 µg/L, respectively. An additional negative control for PAR (0 µg/L) was also included in the experimental design. Water flux was constant in order to maintain optimal conditions. Animals were fed *ad libitum* three times a week with commercial fish food (Activpet^®^, Lisbon, Portugal). During exposure, dissolved oxygen and temperature were measured using a multiparameter probe (YSI, 556 MPS) for validation purposes. After 28 days of exposure, animals were anesthetized using ice, until immobilization, and sacrificed by being cut into small pieces with a scalpel. Animals were divided into small portions, placed into microtubes, and frozen in liquid nitrogen (−196 ${}^{{}^{\circ}}$C) to prevent enzyme degradation. Thereafter, samples were stored at −80 ${}^{{}^{\circ}}$C for later biochemical assays.

### 2.5. Tissue Processing

Samples for catalase (CAT), glutathione S-transferases (GSTs), and lipid peroxidation (TBARS) determinations were homogenized with 1.0 mL of phosphate buffer, 50 mM, pH 7.0, with Triton X-100, sonicated (Brandson Sonifier 250) for 30 s, and centrifuged (TermoFisher Megafuge 8R) at 15,000×*g* for 10 min at 4 ${}^{{}^{\circ}}$C [70]. For acetylcholinesterase (AChE) quantification, homogenization was performed in 1.0 mL of phosphate buffer, 100 mM, pH 7.2. Afterwards, sonication and centrifugation (3330×*g* for 3 min at 4 ${}^{{}^{\circ}}$C; TermoFisher Megafuge 8R) were performed [71]. Cyclooxygenase (COX) used 100 mM Tris-HCl with 1 mM EDTA in a pH 7.8 buffer, followed by sonication and centrifugation at 10,000× *g* for 15 min at 4 ${}^{{}^{\circ}}$C [72]. For all biomarker determinations, 9 organisms (n = 9) were used. All spectrophotometric readings were performed on a Thermo Scientific Multiskan EX spectrophotometer (using Ascent Software 2.6).

### 2.6. Biomarker Quantification

CAT activity was measured as described by Aebi [73]. Previously homogenized samples were incubated with 30 mM H_2_O_2_, and absorbance was monitored spectrophotometrically at 240 nm. CAT activity was expressed as µmol of H_2_O_2_ consumed per minute, per milligram of protein.

GSTs activity was determined according to Habig et al. [74]. The conjugation of a glutathione solution (10 mM) with the substrate 1-chloro-2,4-dinitrobenzene (CDNB; 60 mM) formed a thioether that was followed spectrophotometrically by an increase in absorbance at a wavelength of 340 nm. Enzymatic activity was expressed as nmol of thioether produced per minute, per milligram of protein.

TBARSs were expressed as malondialdehyde (MDA) equivalents, calculated using an extinction coefficient of 1.569 × 10 ^5^ M^−1^cm^−1^. This methodology is based on the reaction of compounds such as malondialdehyde (formed by the degradation of membrane lipids by a free radical attack) with a thiobarbituric acid (TBA) solution (70 mM) [75] and was determined spectrophotometrically at 535 nm. TBARS levels were expressed as nmol of MDA per mg of protein.

Scaps et al. [76] showed that the predominant form of cholinesterase in *H. diversicolor* is AChE. Therefore, AChE activity was quantified according to Ellman et al. [77]. This method is based on the rate of acetylthiocholine degradation, by the AChE activity, into acetate and thiocholine. This last product complexes with dithionitrobenzoic acid (DTNB) creating a product whose formation can be monitored by measuring the absorbance at 412 nm. Previously homogenized and centrifuged samples were incubated with a reaction solution of acetylthiocholine (0.48 mM) and DTNB (0.32 mM). AChE activity was expressed in nmol of complex formed per minute, per milligram of protein.

Cyclooxygenase activity was measured according to a method performed by Petrovic and Murray [72], where COX converts arachidonic acid in prostaglandin G2 (PGG2). In the presence of {N’,N’,N’,N’-tetramethyl-p-phenylenediamine (TMPD), it reduces itself to its alcohol prostaglandin H2 (PGH2). TMPD oxidation is followed spectrophotometrically at 590 nm. Previously homogenized and centrifuged samples were incubated with a reaction solution of TMPD (100 µM) and arachidonic acid (10µM). COX activity was expressed in nmol of TMPD molecules oxidized per minute, per milligram of protein.

Protein concentration of samples was quantified according to the Bradford method [78] at 595 nm using gama-globulin as a standard (1 mg/mL).

### 2.7. Statistical Analysis

Data were tested for normality (Shapiro–Wilk test) and homogeneity of variances (Levene test). A two-way analysis of variance (Two-Way ANOVA: PAR concentration X pH value), followed, if needed (*p* < 0.05), by a Dunnett’s test, was performed to determine differences between experimental treatments and control groups (pH value of 8.2 and a PAR concentration of 0 µg/L). Statistics were run using the software SPSS v.25, and the level of significance adopted was 0.05.

## 3. Results

Catalase activity

**Figure 1:**
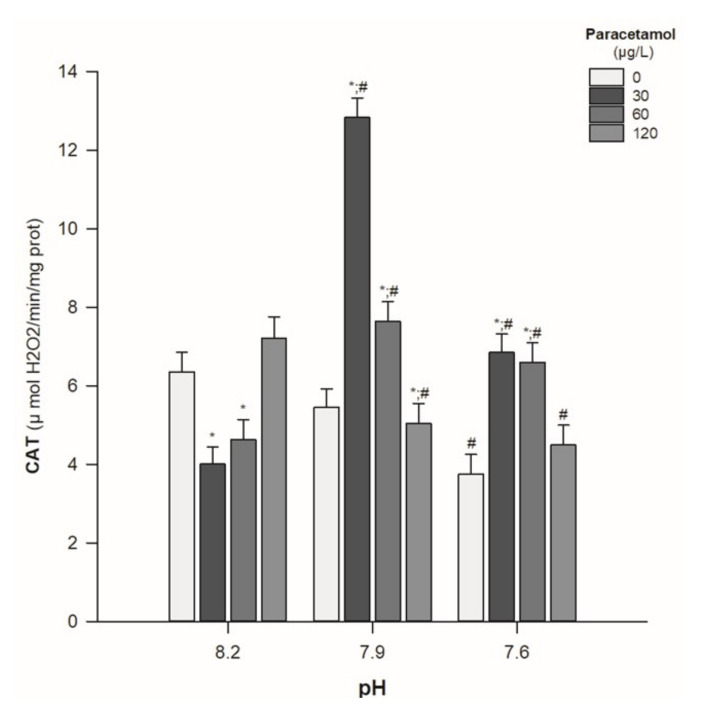
Catalase (CAT) activity in *Hediste diversicolor* exposed to paracetamol (0, 30, 60, and 120 µg/L) under the pH values of 8.2, 7.9, and 7.6. Statistical differences among PAR concentrations (30, 60, and 120 µg/L) and the control group (0 µg/L) are represented with “*”. Statistical differences between pH treatments (7.9 and 7.6) and the pH control group (8.2) within each PAR concentration are represented with “#”. Statistics refer to Dunnett’s tests (following a Two-Way ANOVA). Values are mean ± standard errors.

An impairment in CAT activity was observed in organisms subjected to the two lower PAR concentrations (30 and 60 µg/L) and kept under pH 8.2. On the other hand, CAT activity was generally increased in animals subjected to lower pH values (7.9 and 7.6) at lower PAR concentrations (namely, 30 and 60 µg/L). However, the highest PAR concentration (120 µg/L) showed an opposite trend by decreasing CAT activity in media with the pH values of 7.9 and 7.6 (Table 2; Figure 1). Moreover, there was a significant interaction between PAR concentration and pH value (*p* < 0.05) (Table 2).

GSTs activity

**Figure 2:**
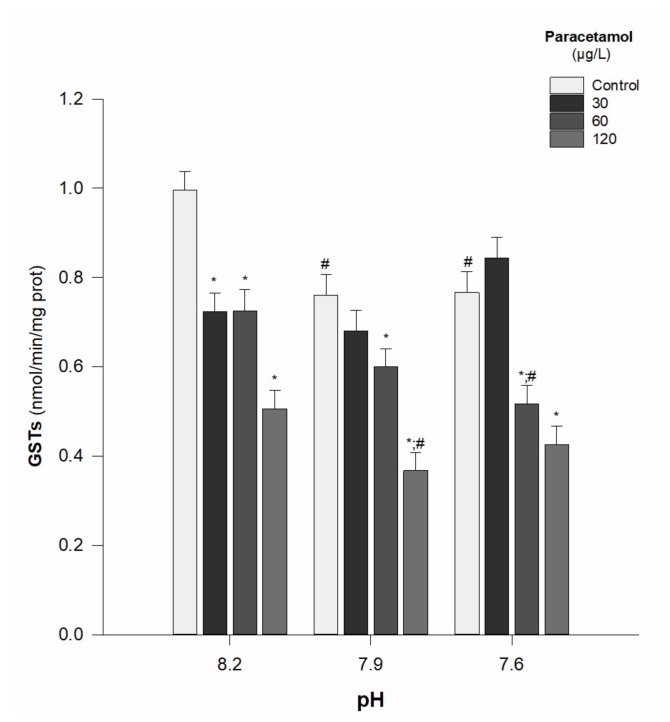
Glutathione S-transferases (GSTs) activity in *Hediste diversicolor* exposed to paracetamol (0, 30, 60, and 120 µg/L) under the pH values of 8.2, 7.9, and 7.6. Statistical differences among PAR concentrations (30, 60, and 120 µg/L) and the control group (0 µg/L) within each pH level are represented with “*”. Statistical differences between pH treatments (7.9 and 7.6) and the pH control group (8.2) within each PAR concentration are represented with “#”. Statistics refer to Dunnett’s tests (following a Two-Way ANOVA). Values are the mean ± standard errors.

There was a general decrease in the GSTs activity along with the increase in PAR concentrations, for all tested pH values (8.2, 7.9, and 7.6). This inhibitory effect was particularly evident for the two higher concentrations (60 and 120 µg/L) of PAR. A GSTs impairment due to the pH was noticeable for animals of the control group, at the pH values of 7.9 and 7.6 (when compared to 8.2) and for those exposed to 60 µg/L and 120 µg/L, at pH 7.6 and 7.9, respectively (Table 2; Figure 2). Moreover, there was a significant interaction between PAR concentration and pH value (*p* < 0.05) (Table 2).

Lipid peroxidation

**Figure 3:**
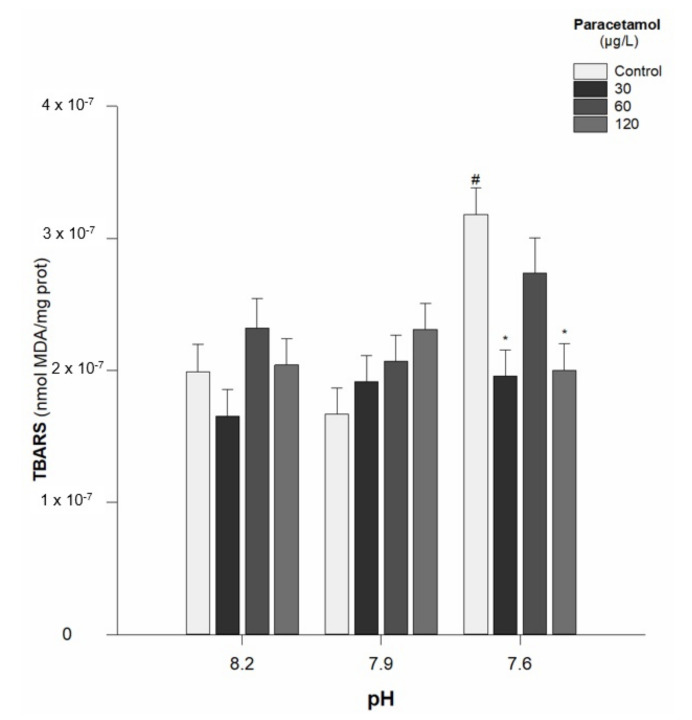
Thiobarbituric acid reactive substance (TBARS) content in *Hediste diversicolor* exposed to paracetamol (0, 30, 60, and 120 µg/L) under the pH values of 8.2, 7.9, and 7.6. Values are the mean ± standard errors. Statistical differences among PAR concentrations (30, 60, and 120 µg/L) and the control group (0 µg/L) are represented with “*”. Statistical differences between pH treatments (7.9 and 7.6) and the pH control group (8.2) within each PAR concentration are represented with “#”. Statistics refer to Dunnett’s tests (following a Two-Way ANOVA). Values are mean ± standard errors.

TBARS concentrations showed no general trend, although a significant increase in lipid peroxidation was observed for the individuals of the control group (0 µg/L) at the lowest pH (7.6). At this same pH, a decrease in TBARS was observed for worms exposed to concentrations of 30 and 120 µg/L (Table 2; Figure 3). In addition, there was a significant interaction between PAR concentration and pH value (*p* < 0.05) (Table 2).

AChE activity

**Figure 4:**
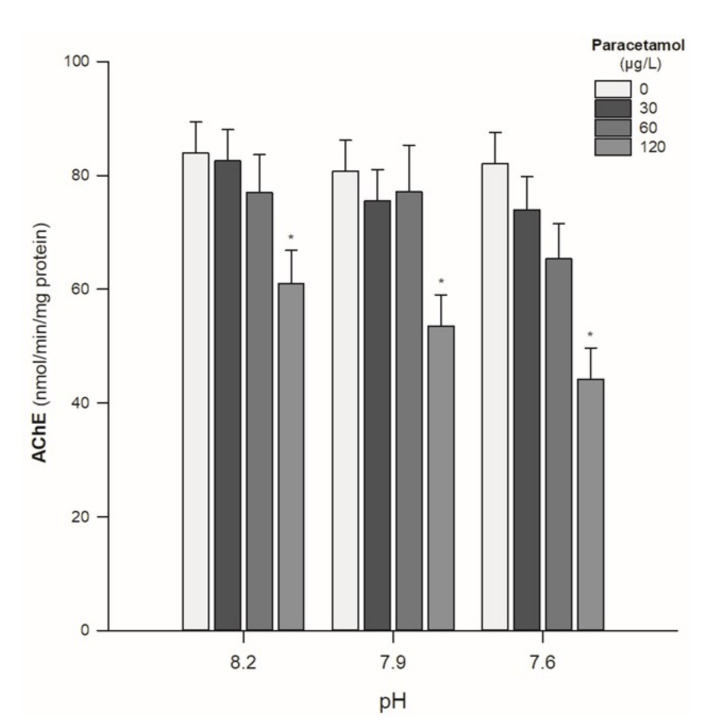
Acetylcholinesterases (AChE) activity in *Hediste diversicolor* exposed to paracetamol (0, 30, 60, and 120 µg/L) under the pH values of 8.2, 7.9, and 7.6. Statistical differences among PAR concentrations (30, 60, and 120 µg/L) and the control group (0 µg/L) are represented with “*”. Statistics refer to Dunnett’s tests (following a Two-Way ANOVA). Values are mean ± standard errors.

Regarding the AChE activity, no effects of pH were observed, regardless of the tested PAR concentrations. However, for all pH values, a significant reduction in AChE was observed for the individuals exposed to the higher PAR concentration (120 µg/L) (Table 2; Figure 4). No interaction was recorded between PAR concentration and pH values (*p* > 0.05) (Table 2).

COX activity

**Figure 5:**
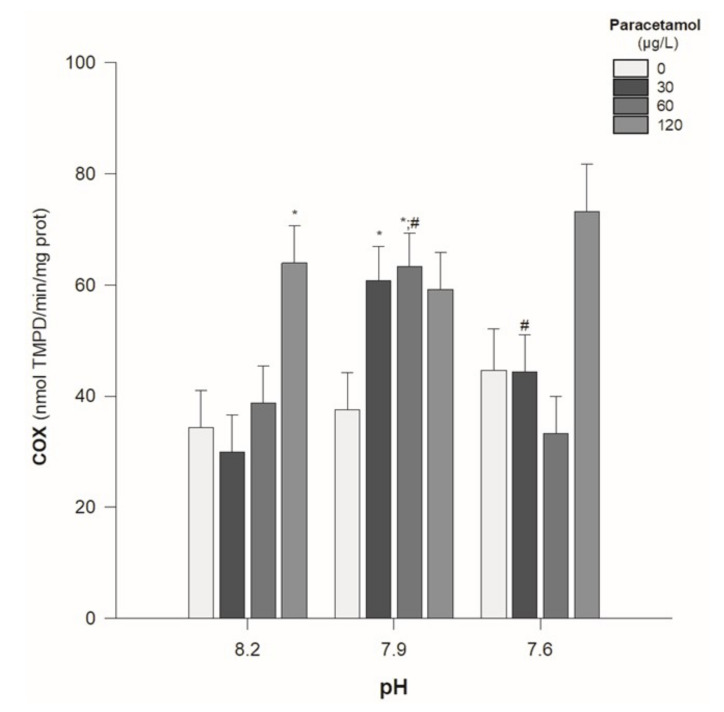
Cyclooxygenase (COX) activity in *Hediste diversicolor* exposed to paracetamol (0, 30, 60, and 120 µg/L) under the pH values of 8.2, 7.9, and 7.6. Statistical differences among PAR concentrations (30, 60, and 120 µg/L) and the control group (0 µg/L) are represented with “*”. Statistical differences between pH treatments (7.9 and 7.6) and the pH control group (8.2) within each PAR concentration are represented with “#”. Statistics refer to Dunnett’s tests (following a Two-Way ANOVA). Values are mean ± standard errors.

COX activity was significantly enhanced in animals exposed to the highest PAR concentration (120 µg/L) under pH 8.2 and in animals exposed to the lower (30 µg/L) and intermediate (60 µg/L) concentrations at the pH value of 7.9 (Table 2; Figure 5). Moreover, there was a significant interaction between PAR concentration and pH value (*p* < 0.05) (Table 2).

## 4. Discussion

To protect against oxidative damages, organisms have developed defensive systems that neutralize reactive chemical species, namely reactive oxygen species (ROS) [79,80]. An important enzymatic defence against hydrogen peroxide (H_2_O_2_) is CAT, since it decomposes this chemical into water and oxygen [81]. In this study, despite the occurrence of significant changes of its activity, CAT did not follow a straightforward pattern according to the increase in PAR concentrations, and no clear or evident trend seemed to result from exposure to different pH levels. It was possible to observe fluctuations of this enzyme´s activity, which were modulated by specific conditions, but no absolute tendency valid for all conditions was reported. This assumption suggests that distinct conditions of PAR concentration and pH yielded different biological outcomes, deserving a deeper discussion. PAR metabolism results in the formation of the highly reactive and oxidant intermediate N-acetyl-p-benzoquinone imine (NAPQI), whose presence may result in oxidative stress [82]. In such a scenario, an increase in CAT activity would have been expected. Nevertheless, H_2_O_2_ is not the only oxidant reactive chemical contributing to the enhancement of oxidative stress involved in the toxicity caused by high amounts of PAR. Other highly reactive oxidant products (that were not measured in this study), such as superoxide anion and NAPQI, may also contribute to the establishment of oxidative stress, affecting endogenous molecules or giving rise to additional ROS [82]. Parolini et al. In [83], it was suggested that PAR toxicity in aquatic organisms (particularly mussels of the species *Dreissena polymorpha*) is dose-dependent, so it is expected that increased amounts of NAPQI may be formed at higher concentrations of PAR. However, from our results, increasing PAR concentrations did not increase CAT activity. In fact, an opposite trend was evidenced in animals exposed to the pH of 7.6, suggesting not only that PAR toxicity is species-specific but that other factors (e.g., water pH) may be also be involved in the modulation of CAT activity. Indeed, a pH of 7.6 (closer to neutrality than the normal sea water pH) may cause an increase in ROS content, augmenting the efficacy of the antioxidant defence system, reflected by a rise of CAT activity, as reflected by our data. This condition has been described in the marine oyster species *Crassostrea virginica* by Tomanek et al. [56]. The authors suggested that this increase in ROS content might have occurred considering two hypotheses: the first suggests that CO_2_ can directly interact with ROS that has already formed, creating additional ROS. The second possibility is that lower pH values may intensify the Fenton reaction, increasing ROS production. This scenario was also evidenced by Sun et al. [84] after subjecting individuals of the marine mussel species *Mytilus edulis* to pH values of 7.7 to 7.1, which are below the normal pH of seawater. This effect of pH by itself seems to support the distinct response obtained for animals exposed to more acidic media, when compared to those conditioned under normal pH values. A general trend showed that animals exposed to these pH values had higher levels of CAT activity in their tissues. It thus seems that pH alone may strongly modulate the extent of the production of ROS that result from the metabolism of specific compounds (such as PAR), increasing the need for the activation of an antioxidant response. Consequently, the worms tested here that were exposed to pH values of 7.6 and 7.9 are likely to have increased their CAT activity in response to the chemical insult caused by PAR metabolism.

Another aspect to be considered is related to the form in which PAR may occur in the aquatic medium. According to the hypothesis of pH partition, and considering that the tested drug is a weak acid (pKa = 9.38; [45]), PAR tends to be in its free, non-ionized form when the media’s pH levels are below neutrality. As pH approaches the acidic range, PAR is likely to occur in its non-ionized free form, being more readily absorbed by living organisms [41]. In this case, PAR is more likely to be absorbed and consequently metabolized and bioactivated into the previously described reactive intermediates, with augmented toxic effects. Considering this possibility, a more evident antioxidant response (with the putative involvement of enhanced CAT activity) would be likely to occur at pH 7.6. In fact, this pattern of increased activities of antioxidant enzymes (namely of CAT) did occur when animals were exposed to pH values below the normal pH of seawater. At normal pH (8.2), there was a decrease in CAT activity for such PAR concentrations. Nevertheless, the effect of increased CAT levels was only significant for worms exposed to low and medium PAR concentrations under more acidic pHs (7.9 and 7.6). Animals exposed to 0 and 120 µg/L of PAR and kept at the lowest pH value (7.6) also showed a similar pattern of enhanced CAT activity. This paradoxical outcome remains to be fully elucidated, but the efficacy of the antioxidant defence system of polychaetes is not restricted to the enzymatic defence system, contrary to what occurs in mammals. This strong difference between mammals and invertebrates, in terms of their antioxidant system, was made evident when analyzing data for all pH levels, but was obtained for organisms exposed to the highest concentrations of PAR. In this case, differences in terms of CAT activity obtained for lower levels of PAR were not reported, and such values were even similar to those measured in control animals. It is possible to suggest that a compensatory response in terms of oxidative stress, involving other mechanisms that are alternatives to CAT (which were not measured in our study), was only triggered at high levels of PAR. In this case, it is possible to hypothesize that a more complex and comprehensive set of antioxidant responses was put in place in worms exposed to the highest PAR concentrations. Despite not being previously shown to occur in aquatic organisms, this trend was reported to occur in other animal models. In fact, short-term stress caused by acute PAR exposure has been shown to occur in rodents by Lores Arnaiz et al. [85], with significant decreases in CAT activity being reported. A plausible confounding factor affecting the interpretation of our data may rely on the presence of polychaetes’ antioxidant pigments, such as carotenoids and biliverdin [86,87,88]. These pigments can act as ROS scavengers, thereby neutralizing ROS resulting from high PAR concentrations, long before CAT activation [86]. In addition, as shown by the study conducted by Pörtner et al. [89], antioxidant pigments can protect against damages caused by pH shifts under CO_2_ acidification, thereby changing the biological responses to other chemicals to which the organisms are being simultaneously exposed.

It is known that PAR is mainly metabolized into non-toxic metabolites by conjugation, particularly with free glutathione (GSH), spontaneously and/or with the intervention of GSTs [82,90]. The data here show a general inhibition of GST activity for almost all pH levels tested, being more evident at higher concentrations of PAR, despite not being statistically significant in all cases. As Manimaran et al. [91] suggested, PAR toxicity can be dose-dependent, and higher doses should theoretically result in more evident effects, with the activation of the metabolic enzymes measured in this study. However, enzyme inhibition may be due to restrictions of the available co-factor that is required for conjugation; that is, in our case, GSH depletion could result in lowered GST activity, as has been suggested by several authors [34,59,92]. In addition, Zhang et al. [58,93] associated a decrease in GST activity, observed in the marine copepod species *Centropages tenuiremis* and *Calanus sinicus* respectively, with the CO_2_-driven acidification of water. The authors suggested that this impairment could be due to a downregulation of GST mRNA biosynthesis caused by increased levels of CO_2_. This outcome has been reported by Todgham and Hofmann [94] in *Strongylocentrotus purpuratus* larvae exposed to a CO_2_ acidification. The CO_2_ acidification of water may compromise four major physiological processes, such as biomineralization, cellular stress response, metabolism, and apoptosis. Other factors that may lead to the reduction of GST activity may be the accumulation of NAPQI, which is likely to result in an increase in ROS content, thus causing cellular damages [95]. Among these damages, one may find direct enzymatic inactivation by denaturation [92,96,97]. GSTs are responsible for the conjugation of NAPQI with GSH [82] and with hydroperoxides resulting from NAPQI oxidation [98]. From the results obtained here, it is clear that pH itself may potentiate the toxic effects of PAR. In fact, in animals exposed to pH values of 7.6 and 7.9, and exposed to both intermediate and higher PAR concentrations, it was possible to observe an additional inhibitory effect of GSTs, when compared to animals subjected to a pH of 8.2 (normal) and simultaneously exposed to the same levels of this drug. This combination of results suggests that the toxicity of paracetamol, whose absorption seems augmented at lower pH values, was enhanced by lower pH levels.

Despite the occurrence of significant effects in terms of the antioxidant defence system of PAR-exposed animals (with alterations of CAT activity), in most animals, no lipid peroxidation was observed. This absence of effects was noticed for worms exposed to all tested PAR concentrations and simultaneously subjected to pH values of 8.2 and 7.9. This can suggest that, despite the occurrence of alterations in antioxidant (CAT) and conjugation metabolism (GST) biomarkers, *H. diversicolor* individuals were not prone to oxidative damage, reinforcing the success of the antioxidant defensive mechanisms that were effectively deployed against this insult resulting from PAR exposure. Similar results were obtained by Giménez and Nunes [99] when exposing *Gibulla umbilicalis* to similar PAR concentrations. Additionally, Liao et al. [100] showed that the exposure of *Patinopecten yessoensis* to acidic conditions (pH = 7.5) was not effective in eliciting peroxidative damages; in conclusion, media pH has very little effect on lipid peroxidation. These results are extremely interesting, since worms kept at the same pH level (but exposed to the highest and lowest concentrations of PAR) evidenced an opposite trend. In fact, these animals had decreased levels of lipid peroxidation in their tissues, suggesting the involvement of alternative mechanisms, namely of the non-enzymatic component of antioxidant defences, as has been discussed.

AChE plays an important role in the nervous system since it prevents continuous neuronal communication by removing the neurotransmitter acetylcholine from the synaptic clefts [101]. This enzyme activity was impaired in organisms exposed to the highest PAR concentrations for all tested pH values. Several authors have found a similar pattern of AChE inhibition in PAR-exposed aquatic organisms within the same µg/L range, namely *Mytilus galloprovincialis* [102], *Anguilla anguilla* [34], and *Ruditapes philippinarum* [37]. These authors suggest that this impairment may occur due to direct enzyme denaturation/inactivation by ROS. In fact, as demonstrated by Delwing-Lima et al. [60], AChE is likely to be oxidized in vivo by ROS in rats, resulting therefore in its hydrolytic inactivation. This deleterious effect of PAR can compromise neural, muscular, and behavioural responses [37]. It thus seems that pH did not contribute to any significant change in the neurotoxic effects caused by PAR exposure, and inhibitory patterns were similar among all conditions. In general terms, PAR concentration seems to have been the most important factor, regardless of the pH values, since higher inhibitory effects were attained at higher levels of exposure, following a dose–response relationship that was valid for all pH conditions.

In addition to antioxidant defence and neurotoxicity, endocrine homeostasis is an important endpoint to establish an animal’s physiological condition. PGs play a key role in the inflammatory process, as they do in homeostatic functions [103]. In most organisms, PGs are synthetized from arachidonic acid by the action of the enzyme COX. COX isoforms (both COX-1 and COX-2) are the main target of the pharmaceutical class of the NSAIDs in mammals [103,104] and seem also to be affected by PAR, but to a lesser extent [52,105,106]. The data obtained here shows no pattern in terms of alteration in COX activity, in animals exposed to all pH levels and/or drug treatments. Nevertheless, Maranho et al. [52] registered a decrease in COX activity when exposing this species to NSAID-contaminated sediments. Therefore, since PAR is a weak inhibitor of both COX isoforms, exposure seems not to have been absorbed in the required amounts to sustain significant impairments of these enzymes, rendering this species, under the adopted conditions, unresponsive to this drug in terms of this specific parameter.

It seems that PAR exposure, under varying but ecologically likely pH conditions, can result in antioxidant stress responses, namely through GST impairment, despite the absence of noteworthy alterations in TBARS content. This finding has a strong ecological significance, since it is possible to hypothesize that PAR exposure, namely under conditions of pH more close to neutrality (as those that are predicted to occur in the future), may compromise the physiology of environmentally exposed organisms. Such alterations are likely to jeopardize this species’ ecological role by impairing behavioral traits, such as movement and sediment mixing, contributing to the hypoxia of estuarine sediments in which this species occurs. Furthermore, pH seems to play a role in the PAR absorption and toxicity in this species, as reflected by the modulation of the effects of this compound (namely reflected changes in the glutathione conjugation pathway, and in the antioxidant mechanism of H_2_O_2_ detoxification). Despite not having by itself played a major role in the toxic effects reported, pH did seem to modulate the toxicity of this drug, which was mostly dependent on the levels of paracetamol to which animals were exposed. It should also be noted that some of the initially selected biomarkers were not fully responsive to distinct pH values and combined paracetamol exposures. Despite having been selected based on the known effects and metabolic routes of paracetamol (as described in the introduction), some of these tools and pathways were not significantly changed, or were altered following coherent patterns, suggesting that alternative analytical tools should be used in the future to assess the interaction of drugs with varying pH levels. Consequently, further studies with an alternative and complimentary set of biomarkers are needed along with a quantification of paracetamol or the enzymes’ metabolites (namely, COX products) in the organisms to fully assess the effect of seawater acidification on the absorption, bioconcentration, and combined effects of acidic or basic pharmaceuticals. 

## Figures and Tables

**Table 1:** Multiple reaction monitoring transitions and mass spectrometry parameters.

Compound	Retention Time	Mass Transition	Cone Voltage	Collision Energy
Acetaminophen	2.08 ± 0.05	152.2 > 65 ^a^	25	25
		152.2 > 110.3 ^b^	25	15
Acetaminophen-D4	2.08 ± 0.05	156.2 > 114.2	25	15

^a^ Quantifier ion. ^b^ Qualifier ion.

**Table 2:** Two-way ANOVA results testing the effects of drug concentration and pH values on biochemical parameters of Hediste diversicolor exposed for 28 days to 0, 30, 60, and 120 µg/L of PAR at control pH (8.2), intermediate pH (7.9), and low pH (7.6).

		Concentration	pH	pH × Concentration	Total
	DF	3	2	6	97
GSTs	F	50.174	10.487	3.663	
	*p*-value	<0.0001	<0.0001	<0.0001	
	DF	3	2	6	86
CAT	F	12.474	23.228	27.406	
	*p*-value	<0.0001	<0.0001	<0.0001	
	DF	3	2	6	100
TBARs	F	3.795	6.622	4.246	
	*p*-value	0.013	0.002	<0.0001	
	DF	3	2	6	95
AChE	F	16.166	2.287	0.437	
	*p*-value	<0.0001	0.065	0.952	
	DF	3	2	6	58
COX	F	7.705	4.42	2.912	
	*p*-value	<0.0001	0.020	0.017	

## Data Availability

Not applicable.
